# A novel colorimetric immunosensor based on silk cocoon membrane-integrated ELISA for treponemal antibody detection

**DOI:** 10.1128/spectrum.00094-25

**Published:** 2025-07-22

**Authors:** Hongmei Wang, Yizhi Huang, Qi Xiao, Yezhou Chen, Yujue Wang, Jingjing Tian, Yuqing Cai, Longhai Tang, Shaohua Ding, Shengbao Duan

**Affiliations:** 1 Suzhou Institute of Biomedical Engineering and Technology, Chinese Academy of Sciences 681045, Suzhou, China; 2 Department of Biomedical Sciences, City University of Hong Kong, Hong Kong, China; 3 Suzhou Blood Center, Suzhou, China; 4 Jinan Guoke Medical Technology Development Co., Ltd 681014 , Jinan, China; 5 Blood Transfusion Department, China-Japan Union Hospital of Jilin University 628698 , Changchun, China; Ascension St John Hospital, Detroit, Michigan, USA

**Keywords:** silk cocoon membrane, immunosensor, colorimetric, ELISA, treponemal antibodies

## Abstract

The global rise in syphilis incidence and the challenges of early diagnosis highlight the urgent need for convenient diagnostic tools. In this study, we present a colorimetric immunosensor for the detection of *Treponema pallidum* antibodies (TP-Ab) using a natural silk cocoon membrane (SCM) as the substrate, based on the principle of enzyme-linked immunosorbent assay (ELISA). The TP monoclonal antibody 3H12 and the chimera antigen TP99, comprising epitopes TP15, TP17, and TP47, were sequentially coupled to functionalized SCM to form the SCM-based TP-Ab immunosensor. The detection was quantified by color extraction, and the Corrected *I*_red_ values were used as a judgment parameter. The TP-Ab immunosensor demonstrated a detection limit of 0.48 mIU/mL, lower than the 0.68 mIU/mL limit of commercial microplate ELISA kits. Furthermore, it exhibited no cross-reactivity with antibodies against other pathogens. Testing with a reference serum panel yielded 100% concordance with the reference results. Comprehensive statistical analysis of the Corrected *I*_red_ values from the reference serum panel and banked samples indicated an optimal cutoff value of 0.0921, achieving 100% sensitivity, 97.96% specificity, and high overall accuracy, with an area under the curve of 0.9995 and a 95% confidence interval of 0.9979–1.00. Moreover, a statistically significant correlation with microplate ELISA was observed (*r* = 0.9217, *P* < 0.0001). This reliable ELISA-integrated colorimetric immunosensor, featuring a three-dimensional porous structure and inherent bioactivity, contributes to improving the detection performance and provides a rapid and convenient diagnostic solution for syphilis. It is particularly well-suited for resource-limited settings where on-site testing is critical.

**IMPORTANCE:**

Syphilis remains a major global public health concern, particularly in resource-limited settings where early and accurate diagnosis is often hindered by technical and economic barriers. Here, we report a novel treponemal antibody (TP-Ab) immunosensor based on natural silk cocoon membrane (SCM), a biocompatible and porous material with intrinsic bioactivity. By integrating enzyme-linked immunosorbent assay principles and a recombinant TP15-17-47 chimera antigen, the SCM-based immunosensor enables colorimetric detection of TP-Ab with high specificity and visual readout. Unlike traditional methods that require complex instrumentation or expensive reagents, this platform offers a low-cost, portable, and easy-to-use alternative suitable for on-site or point-of-care testing. The findings highlight the potential of silk-derived biosensors in a promising direction for syphilis screening technologies.

## INTRODUCTION

Syphilis, caused by *Treponema pallidum* (TP), is a highly contagious and systemic sexually transmitted infection (STI). It poses a serious threat to public health due to its potential to damage multiple organ systems, including the nervous, cardiovascular, digestive, and urinary systems (1). In recent years, the global incidence of syphilis has been rising steadily, prompting widespread concern as a major public health issue. According to the World Health Organization, approximately 5.6 million new cases of syphilis are reported annually, with the majority occurring in low- and middle-income countries (2, 3). Early and accurate diagnosis is crucial for controlling syphilis and reducing its incidence, but diagnostic challenges persist due to the disease’s complex clinical presentation.

While direct diagnostic methods, such as pathogen microscopy and molecular biology techniques, can facilitate early detection of syphilis (2, 4), their use is constrained by limitations in performance, availability, and cost. Consequently, serologic testing remains the primary diagnostic approach (5, 6). Serological tests for syphilis are broadly categorized into non-treponemal and treponemal antibody detection methods, both of which are commonly used for screening and confirmation (7, 8). Non-treponemal tests, such as the venereal disease research laboratory test, toluidine red unheated serum test (TRUST), and rapid plasma reagin test, are effective for large-scale initial screenings. However, these tests often lack specificity due to their reliance on non-specific antigens, including cardiolipin, lecithin, and cholesterol (9, 10). Treponemal tests, which offer higher specificity and sensitivity, include the *Treponema pallidum* particle agglutination assay (TPPA), fluorescent treponemal antibody-absorbed (FTA-ABS) test, western blot (TP-WB), chemiluminescence analysis (TP-CLIA), enzyme-linked immunosorbent assay (TP-ELISA). While TPPA, FTA-ABS, and TP-WB offer reliable results, their high cost, operational complexity, and limited automation impede their routine use in clinical practice. Automated methods, such as TP-ELISA and TP-CLIA, provide objective and accurate results, making them more suitable for large-scale sample screening. However, these methods remain impractical for small-scale testing or emergency use, as they require longer processing time (11–13). Additionally, their reliance on specialized lab facilities, skilled technicians, and costly reagents limits their accessibility in resource-limited settings, such as rural areas in developing countries. Given this, the development of alternative rapid treponemal tests suitable for point-of-care deployment could help limit the spread of syphilis by enabling immediate intervention, which presents a critical challenge.

Biosensor-based diagnostic assays have emerged as promising alternatives for diagnosing STIs, offering greater simplicity and faster results compared to conventional methods. Several biosensor platforms have been explored for treponemal antibody detection, including electrochemical sensors, surface plasmon resonance immunosensors, lateral flow biosensors employing gold nanoparticles or quantum dots, and vertical filtration biosensors utilizing paper membranes (14–19). Despite their potential, many biosensors depend on specialized substrates, limiting their practical application in clinical settings. Recently, silk, a natural polymer, has gained attention as a versatile material for biomedical applications due to its excellent biocompatibility, chemical flexibility, and mechanical robustness (20–24). While regenerated silk matrices have been used for sensor applications, their preparation involves complex procedures that hinder large-scale production. Alternatively, natural silk fibers or membranes can be directly used as biosensor substrates, offering a simpler approach without the need for regeneration (25, 26). In our previous work, we demonstrated that natural silk cocoon membrane (SCM), with its free-standing multilayered structure, porosity, and inherent bioactivity, serves as an effective immunosensing platform. The SCM-based platform was shown to enable rapid red blood cell typing in whole blood with superior analytical performance and excellent thermal stability (27, 28).

In this study, we developed a colorimetric immunosensor using SCM as the substrate to detect TP-Ab targeting three highly immunogenic antigens: TP15, TP17, and TP47. Previous studies have shown that recombinant TP antigens improve the performance of treponemal tests, with TP-Ab against TP15, TP17, and TP47 detectable as early as 2–4 weeks post-infection, earlier than non-treponemal antibodies (29–31). Building on these findings, we employed a recombinant chimera TP antigen containing epitopes from TP15, TP17, and TP47 as the recognition element. The antigen was immobilized onto the SCM-based substrate to form a TP-Ab immunosensor, integrated with ELISA principles for colorimetric detection. The resulting immunosensor enables accurate, convenient, and visual detection of TP-Ab without the need for sophisticated equipment or specialized techniques.

## MATERIALS AND METHODS

### Materials and samples

Silk cocoons from *Bombyx mori* were sourced from a local market. The chimera of TP antigens containing epitopes from TP15, TP17, and TP47 (referred to as TP99), along with horseradish peroxidase (HRP)-conjugated TP antigens (HRP-TP mix antigen), and the TP47 antigen and HRP-labeled TP47 (HRP-TP47), were purchased from Fapon Biotech (China). The 3,3′,5,5′-Tetramethylbenzidine (TMB) substrate was obtained from Sigma-Aldrich (USA). Standard reference sera—anti-HBV serum (9 mIU/mL), anti-HCV serum (48 mIU/mL), anti-HIV-1 serum (48 mIU/mL), along with a reference serum panel containing 40 samples, one positive control (6 mIU/mL), one negative control (matrix liquid, 0 mIU/mL), and the standard TP-Ab serum at 3, 6, 12, and 21 mIU/mL concentrations, were sourced from Beijing Conchestan Biotechnology Co., Ltd., China. The detailed information about the reference serum panel and the standard TP-Ab serum is shown in Fig. S1. Treponemal antibody ELISA kits were purchased from Beijing Wantai BioPharm, China (Cat. No. WT-5296). Mouse anti-silk cocoon mAbs (MAS-2H3), goat anti-mouse IgG (GAM-IgG), and mouse anti-TP mAbs (3H12, specific for TP47 antigen) were developed and characterized in-house. A total of 58 banked serum samples, including 30 TP-positive and 28 TP-negative samples, were examined and confirmed using the TRUST and TP-ELISA. These banked samples were kindly provided by the Suzhou Blood Center between January 2024 and August 2024.

### Fabrication of the SCM-based TP-Ab colorimetric immunosensor

**Fig 1:**
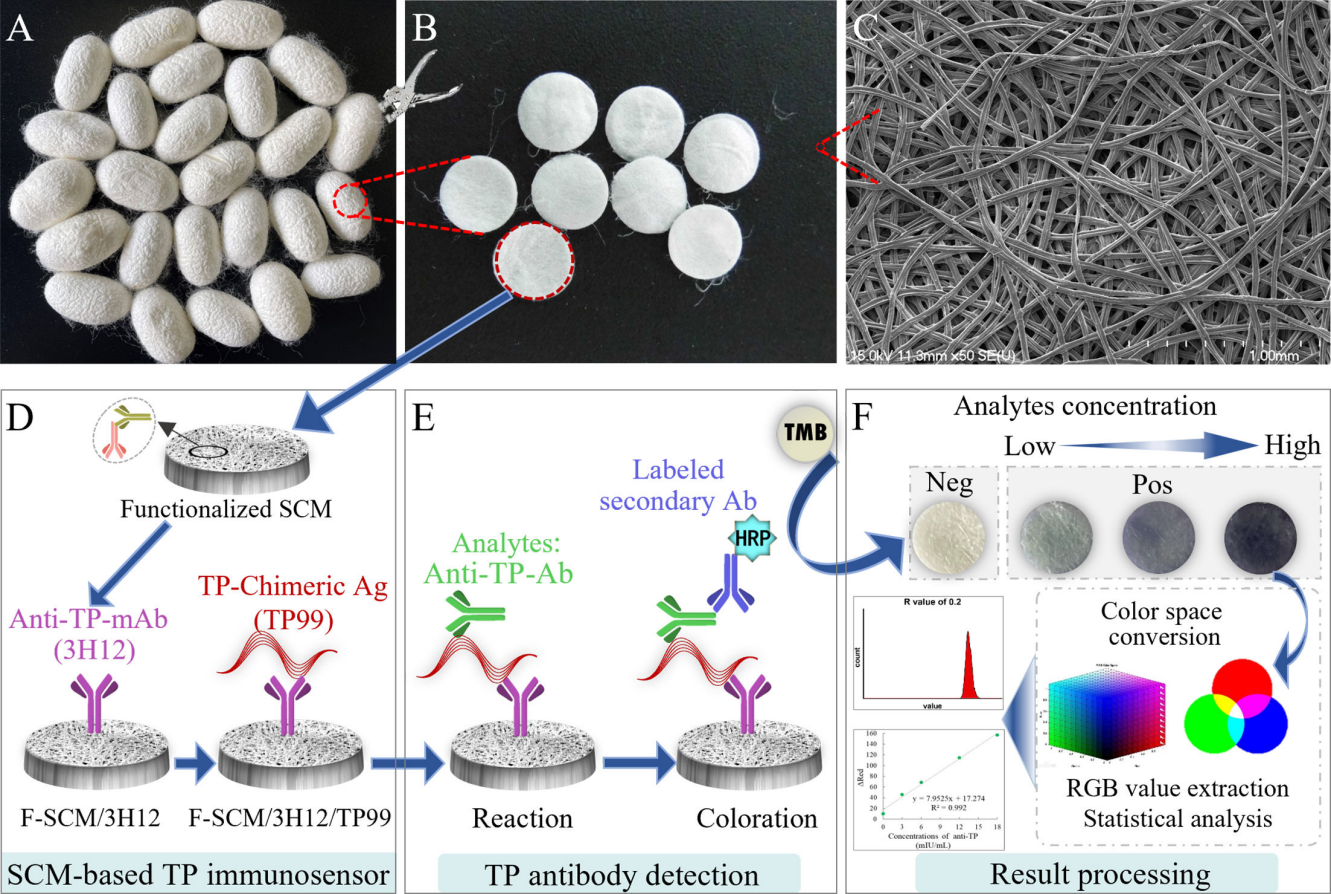
Fabrication and detection procedure of the SCM-based TP-Ab colorimetric immunosensor. (**A**) Natural silk cocoons. (**B**) SCM discs derived from silk cocoons. (**C**) SEM image showing the microporous structure of a typical SCM. (**D**) Schematic representation of the fabrication process for the SCM-based TP-Ab immunosensor (F-SCM/3H12/TP99). (**E**) Detection principle for TP-Ab, and (**F**) the result processing and statistical analysis.

The functionalized SCMs (F-SCMs) were prepared as described in our previous work (27, 28). Briefly, silk cocoons (Fig. 1A) were punched into round discs (8 mm in diameter, Fig. 1B) using a hole puncher, retaining their natural interconnected microporous structure, which was characterized using scanning electron microscopy (SEM) (Fig. 1C). Following pretreatment, the SCMs retained their natural bioactivity and were sequentially coupled with MAS-2H3 and GAM-IgG to form F-SCMs through specific directional immunoaffinity binding. The 3H12 mAb was then immobilized onto the F-SCMs by binding to GAM-IgG, followed by overnight incubation at 4°C. Then, the capture antigen TP99 chimera was immobilized onto the F-SCM/3H12 complexes to serve as the recognition element, completing the formation of the immunosensor membrane (F-SCM/3H12/TP99) (Fig. 1D). After two PBS washes, the membranes were blocked with 5% skim milk in PBS at 37°C for 2 h to prevent nonspecific binding. Finally, the membranes were washed with PBS and air-dried, completing the fabrication of the SCM-based TP-Ab immunosensor.

To optimize 3H12 concentration for immobilization, gradients ranging from 0 to 80 µg/mL (0, 0.2, 1, 5, 10, 20, 40, and 80 µg/mL) were incubated with F-SCMs overnight at 4°C, using 200 µL per disc. The binding efficiency of 3H12 was evaluated via colorimetric reactions using HRP-conjugated TP47 antigen. Once the optimal concentration of 3H12 was determined, the ideal concentration of the TP99 was further confirmed. TP99 concentrations of 0, 1.25, 2.5, 5, and 10 µg/mL were incubated with F-SCMs/3H12 complexes overnight at 4°C, using 200 µL per disc. The optimal TP99 concentration was identified by testing a standard anti-TP serum series (0, 3, 6, 12, and 21 mIU/mL) with HRP-TP mix antigen, and the strongest colorimetric signal was used to confirm the optimal concentration.

### Detection procedure for the SCM-based TP-Ab colorimetric immunosensor

The SCM-based TP-Ab immunosensor was employed in a vertical flow-through ELISA format. The SCM discs were placed on top of 2 cm × 2 cm absorbent pads and prewetted with 50 µL of PBS. Then, 50 µL of sample was added to each disc and incubated at 37°C for 15 min in a water bath. Following this, 100 µL of HRP-TP mix antigen was applied and incubated at 37°C for another 15 min. The discs were then rinsed four times with PBST (PBS + 0.05% Tween-20) on the absorbent pad. Next, 100 µL of TMB substrate was applied to initiate the colorimetric reaction, which proceeded for 5 min at 37°C. The reaction was halted by washing the discs with distilled water. Positive results were indicated by a blue color on the membrane, while negative results retained the natural white color of the SCM. The colorimetric intensity was quantified using ImageJ software. The detailed principle of the detection and the result processing are illustrated in Fig. 1E and F.

### Analytical performance of the SCM-based TP-Ab colorimetric immunosensor

The linear range of detection for the SCM-based TP-Ab immunosensor was evaluated by testing a series of anti-TP Ab serum concentrations: 0, 0.125, 0.25, 0.5, 1, 2, 3, 6, 12, and 21 mIU/mL. The concentrations from 0.125 to 2 were prepared by serial dilution of the 3 mIU/mL standard anti-TP Ab serum. The limit of detection (LoD) of the TP-Ab immunosensor was calculated by three times the standard deviation of the blank signal divided by the slope of the calibration curve at low analyte concentrations (32). This approach reflects the lowest concentration that can be reliably distinguished from background and is widely accepted for evaluating analytical sensitivity. For comparison, a commercial microplate-based ELISA was performed as a reference method, and its optical density in 450 nm (OD_450_) values was analyzed according to the manufacturer’s instructions.

To evaluate the cross-reactivity of the SCM-based TP-Ab immunosensor, standard reference sera—anti-HBV serum (9 mIU/mL), anti-HCV serum (48 mIU/mL), anti-HIV-1 serum (48 mIU/mL), anti-TP serum (6 mIU/mL)—and a negative control (matrix liquid, 0 mIU/mL) were tested. This evaluation aimed to verify the specificity of the immunosensor and confirm the absence of cross-reactivity with antibodies of common coinfection diseases.

The reference serum panel of 40 samples, alongside a positive and a negative control, was utilized to assess the accuracy of the SCM-based TP-Ab immunosensor compared to the official outcomes. Meanwhile, the 58 banked samples comprising 30 positives and 28 negatives were tested by the SCM-based TP-Ab immunosensor and the commercial microplate-based ELISA. Here, two approaches were used to estimate the cutoff value for classifying samples as positive or negative (32–34). First, the crude cutoff value used for classifying positive versus negative results in the reference serum panel was determined according to conventional methodology, defined as the Corrected *I*_red_ mean of the confirmed 28 negative samples in the banked sample plus three times their standard deviation. Then, a ROC curve analysis was performed using MedCalc software (MedCalc Software Ltd, Belgium) based on results from a total of 100 samples (40 samples and alongside a positive and a negative control of the reference serum panel and 58 banked samples). All data were subjected to a comprehensive statistical analysis to further assess the accuracy and consistency.

### Imaging, color extraction, data curation, and statistical analysis

Color changes on the SCM discs were recorded using a smartphone camera (Apple iPhone 15, MTLJ3CH/A). Images were captured in a well-lit environment during the day without the use of external lighting or flash. The recorded images were subsequently processed for colorimetric quantification, where the surface colors of the SCM discs were analyzed to extract red, green, and blue (RGB) values. Histogram analysis of the RGB channels was conducted using ImageJ software (NIH) (Fig. S2) (35). To minimize errors caused by variations in capture conditions or false positives resulting from darker imprints at the substrate edges due to liquid evaporation, a uniform selective area was defined for image analysis (36). The approach effectively excluded dark rings and irregular patches, ensuring accurate pixel value representation by averaging intensity values across a uniformly distributed area. Corrected RGB intensities (Corrected *I*_RGB_) for red (Corrected *I*_red_), green (Corrected *I*_green_), and blue (Corrected I_blue_) channels were calculated using equation 1.


(1) $$Corrected\;I_{RGB}=\log_{10}\left(\frac{RGB\_blank}{RGB\_sample}\right).$$

ROC curve analysis was performed to determine the threshold cutoff values for distinguishing positive from negative samples more accurately and calculate sensitivity and specificity by using the larger sample size. Additionally, the correlation between OD_450_ values from the microplate-based ELISA assay and Corrected *I*_red_ values from the SCM-based colorimetric immunosensor was evaluated using Spearman’s correlation. Data visualization and figure preparation were carried out using GraphPad Prism software (GraphPad 10.1.2).

## RESULTS

### Selection of the optimum color channel

To optimize the performance evaluation of the SCM-based TP-Ab immunosensor, we employed RGB image analysis to identify the most sensitive primary color channel. In digital images, pixel intensity is represented by the red, green, and blue channels. To determine which channel exhibited the highest color variability for our immunosensor, we independently tested and compared each primary color channel.

**Fig 2:**
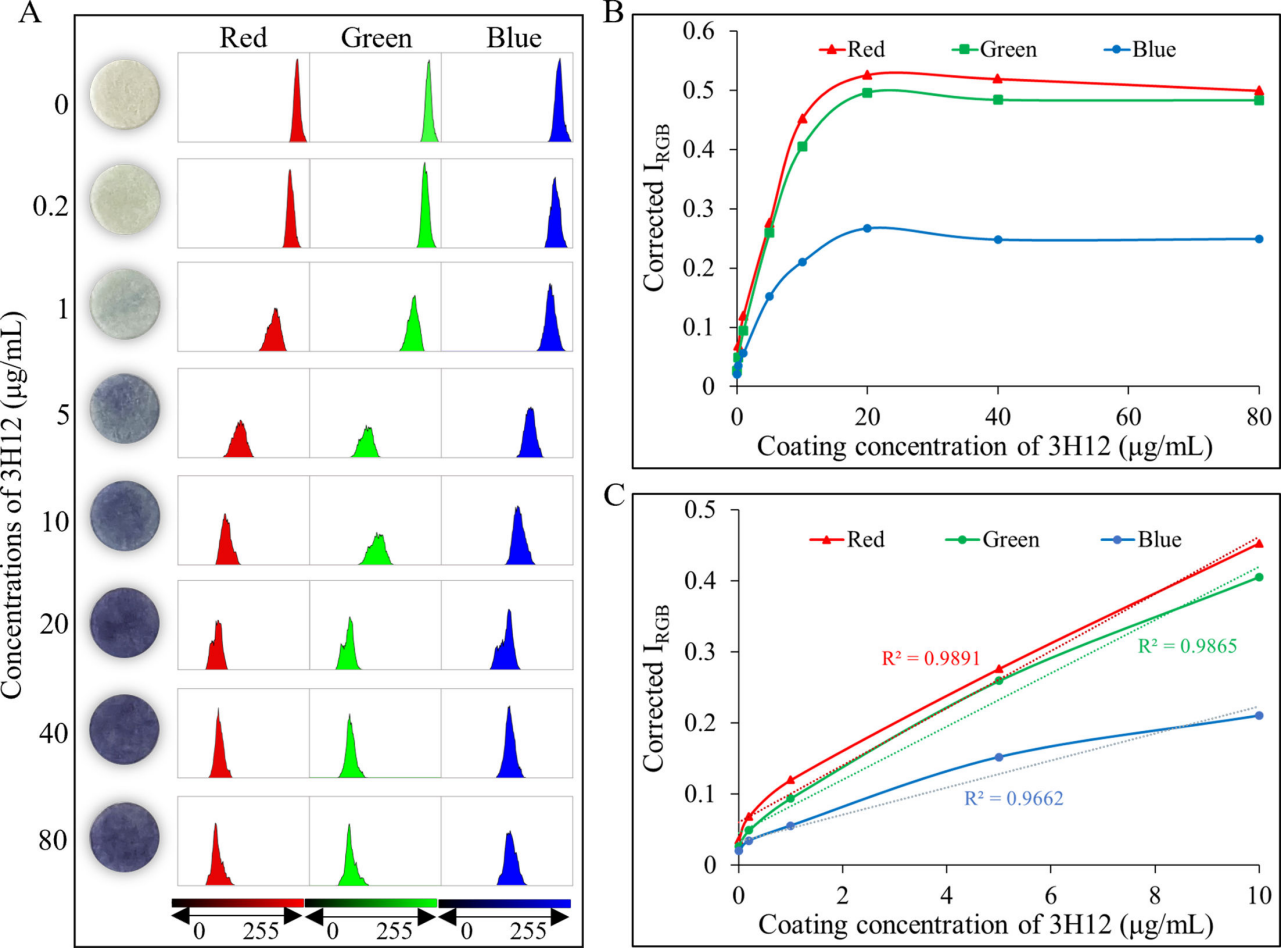
The RGB image analysis to identify the color channel. (**A**) Representative images displaying color changes in the red, green, and blue channels for various 3H12 coating concentrations, with corresponding histograms illustrating intensity variations. (**B**) Linear trends of Corrected *I*_red_, *I*_green_, and *I*_blue_ versus 3H12 coating concentrations (0–80 μg/mL). (**C**) Linear correlation between Corrected *I*_RGB_ and 3H12 coating concentrations in the 0–10 μg/mL range for the Corrected *I*_red_, *I*_green_, and *I*_blue_ channels.


As shown in Fig. 2A and B, the Corrected *I*_red_, Corrected *I*_green_, and Corrected *I*_blue_ channels were measured across a 3H12 concentration range of 0–80 µg/mL, and concentration-dependent data were plotted. Notably, the red channel demonstrated the highest color variability, indicating superior signal sensitivity. Consequently, in all subsequent experiments, Corrected *I*_red_ was selected as the primary analytical signal, calculated using equation 2. Here, Corrected *I*_red_ represents the mean pixel intensity of the red channel obtained from the selected area of the SCM-based TP-Ab colorimetric immunosensors.

(2) $$Corrected\;I_{red}=\log_{10}\left(\frac{R\_blank}{R\_sample}\right).$$

### Fabrication of the SCM-based TP-Ab colorimetric immunosensor

Building upon the F-SCMs developed in our previous research (28), we constructed the SCM-based TP-Ab immunosensor based on the already prepared F-SCMs through further immunological affinity coupling. First, 3H12 antibodies were immobilized onto the prepared F-SCMs, creating F-SCMs/3H12. TP99 antigen was then introduced to specifically bind with 3H12, acting as the capture antigen for detecting TP-Ab.

The intensity of the reaction for various concentrations of immobilized 3H12 was assessed (0, 0.2, 1, 5, 10, 20, 40, and 80 µg/mL), and the results showed that the reaction intensity peaked at 20 µg/mL, with saturation occurring at concentrations above 40 µg/mL (Fig. 2B). The optimal concentration of immobilized 3H12 was determined when the Corrected *I*_red_ intensity reached saturation and stabilized. At concentrations below 10 µg/mL, the Corrected *I*_red_ displayed a higher value and a better linear correction with 3H12 concentration (Fig. 2C). Therefore, 20 µg/mL was identified as the optimal 3H12 immobilization concentration for subsequent experiments.

**Fig 3:**
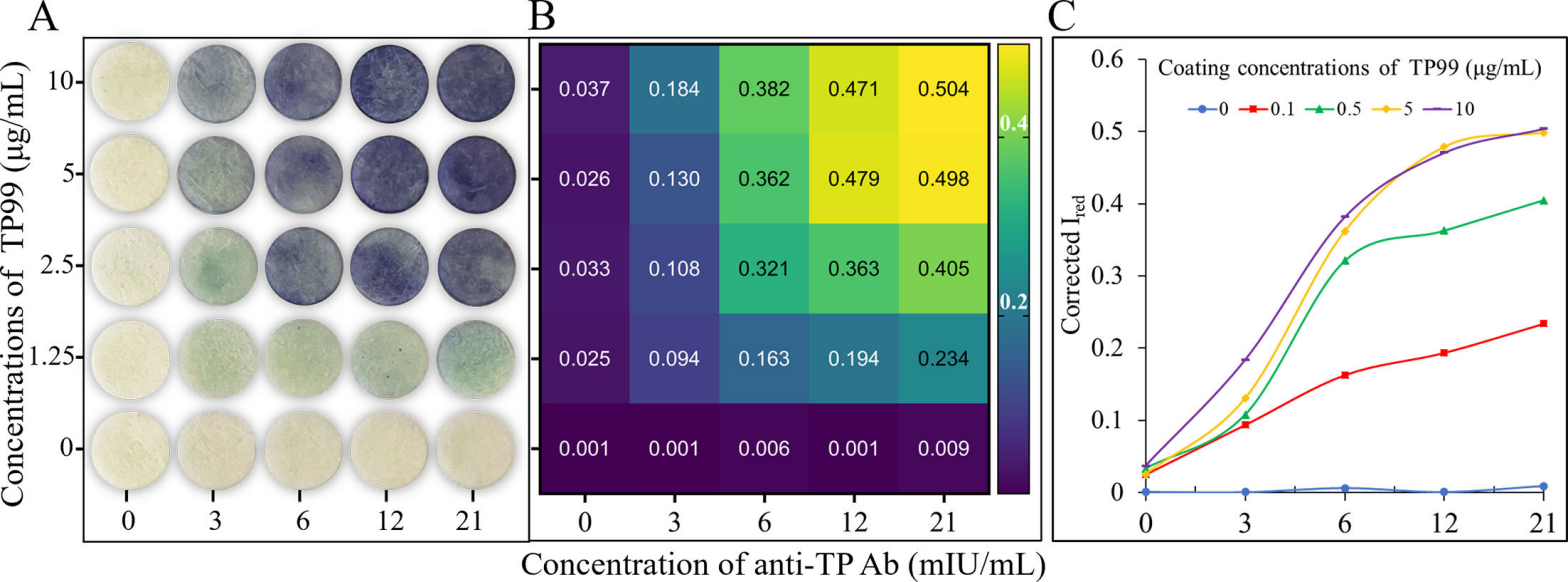
Confirmation of the optimal coupling concentration of TP99 for the preparation of F-SCMs/3H12/TP99. (**A**) Representative image showing colorimetric results for various TP99 coating concentrations. (**B**) Heatmap of the Corrected *I*_red_ signals corresponding to the results in panel **A**. Panels **A** and **B** share the same vertical axis. (**C**) Corrected *I*_red_ signals illustrating the relationship between TP99 coating concentration and TP antibody levels in standard serum. Panels **A–C** share the same horizontal axis.

Subsequently, the TP99 chimeric antigen was coupled to F-SCMs/3H12, creating the SCM-based TP-Ab immunosensor (F-SCMs/3H12/TP99). Various concentrations of TP99 (0, 1.25, 2.5, 5, and 10 µg/mL) were tested alongside standard TP antibody concentration (0, 3, 6, 12, and 21 mIU/mL) to determine the optimal TP99 coupling concentration. The results showed that at 2.5 µg/mL concentrations of TP99, the immunosensor could not effectively detect high antibody levels. However, when the TP99 concentration was at 5 µg/mL, the TP-Ab immunosensor displayed optimal performance, efficiently detecting antibodies and reaching saturation (Fig. 3A through C). As a result, 5 µg/mL was selected as the optimal TP99 concentration for the SCM-based TP-Ab immunosensor.

### Analytical performance of the SCM-based TP immunosensor

**Fig 4:**
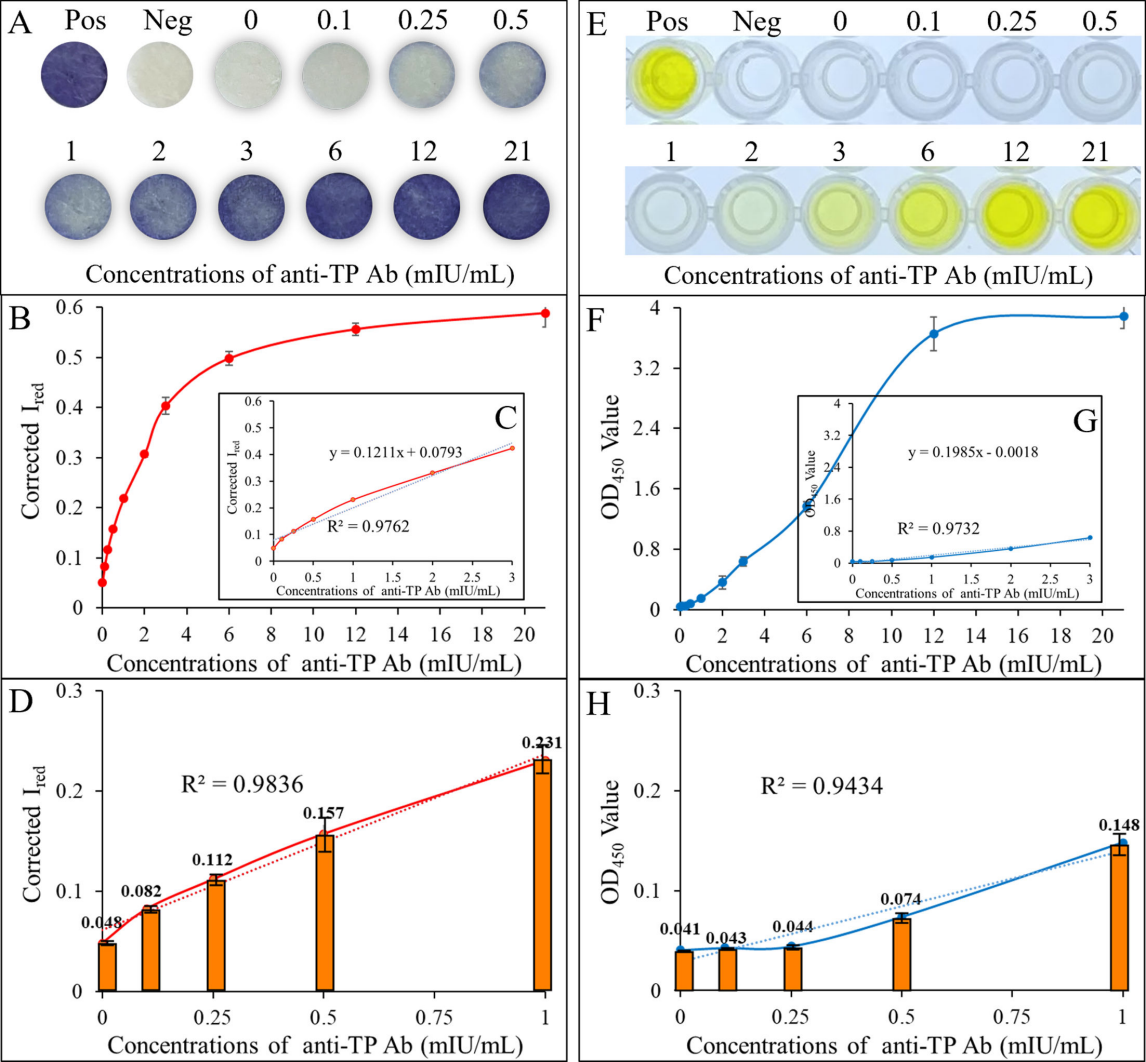
The linear range of detection and LoD for TP-Ab immunosensor and ELISA-based microplate. (**A**) and (**E**) Representative digital images of the TP-Ab immunosensor and microplate ELISA, respectively. (**B and C**) and (**F and G**) Calibration curves comparing Corrected *I*_red_ signals and OD_450_ values for varying TP-Ab concentrations, 0–18 and 0–3 mIU/mL. (**D** and **H**) Calibration curves for low TP-Ab concentrations (0–1 mIU/mL).

A series of standard TP-Ab serum samples with concentrations of 0, 0.1, 0.25, 0.5, 1, 2, 3, 6, 12, and 21 mIU/mL were tested to evaluate the linear range and the limit of detection. The commercially available microplate ELISA kit served as the reference for comparison. The results indicated that with increasing anti-TP antibody concentrations, the TP-Ab immunosensor exhibited a corresponding increase in signal intensity. Signal saturation occurred at a TP-Ab concentration of 12 mIU/mL, which was consistent with the microplate ELISA (Fig. 4A, B, E, and F). Both methods demonstrated a good linear detection range between 0 and 3 mIU/mL, with *R*-squared (*R*^2^) values of 0.9762 and 0.9732, respectively (Fig. 4C and G). However, within the lower concentration ranges of 0–1 mIU/mL, the TP-Ab immunosensor showed a significantly better linear relationship (*R*^2^ = 0.9836) compared to the microplate ELISA (*R*^2^ = 0.9434), which is primarily due to the minimal variation in the OD450 signal of the microplate ELISA between 0 and 1 mIU/mL. In contrast, the TP-Ab immunosensor exhibited more pronounced signal changes, with the Corrected *I*_red_ values ranging from 0.048 to 0.231, compared to the OD_450_ range of 0.048–0.148 for the microplate ELISA. Specifically, the OD_450_ for 0.1 and 0.25 mIU/mL were 0.043 and 0.044, which were close to 0.041 for the blank (0 mIU/mL), making it difficult to distinguish positive results at these low concentrations. Comparatively, the Corrected I_red_ signals for 0.1 and 0.25 mIU/mL were 0.083 and 0.122, respectively, significantly higher than the 0.048 for the blank (Fig. 4D and H). This indicates that the TP-Ab immunosensor has a clear advantage in detecting low antibody concentrations.

Based on these findings, we further analyzed the LoD for both methods using the calibration standard curve derived from the 0–3 mIU/mL anti-TP Ab concentrations. The LoD for each method was calculated by three standard deviations of the blank (0 mIU/mL) divided by the slope of the 0–3 mIU/mL calibration curve. The calculated LoDs were 0.48 mIU/mL for the SCM-based TP-Ab immunosensor and 0.68 mIU/mL for the microplate ELISA. These results demonstrate the superior sensitivity and lower detection limit of the SCM-based TP-Ab immunosensor, which exhibited significant signal variation even at low antibody concentrations.

**Fig 5:**
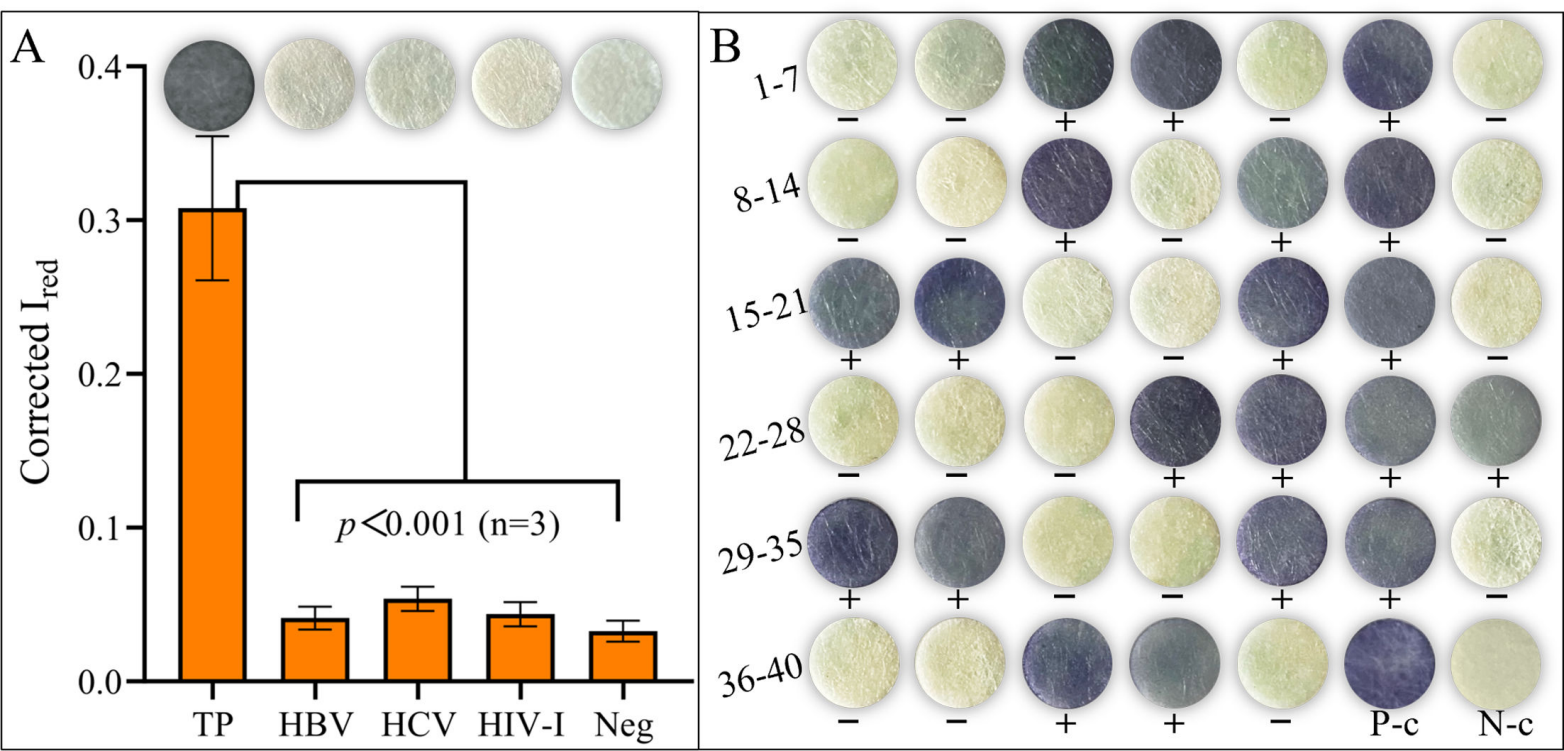
Evaluation of cross-reactivity and detection accuracy of TP-Ab immunosensor. (**A**) Corrected *I*_red_ signals’ colorimetric results of the standard reference serum. (**B**) The results of colorimetric for TP-Ab immunosensor of the reference serum panel, and the reference results tested by treponemal antibody detection using microplate ELISA, represented by + (positive) and − (negative), alongside a positive (P**-**c) and a negative control (N**-c**).

To identify cross-reactivity, the immunosensor was tested with a standard reference serum containing antibodies against TP, HBV, HCV, and HIV-1, and the TP-negative control was detected. The optimized SCM-based TP-Ab immunosensor assay yielded positive results exclusively for the TP standard reference serum, with significantly higher Corrected *I*_red_ signals compared to other samples (*P* < 0.001) as shown in Fig. 5A. Each sample was tested in triplicate. These results confirm the absence of cross-reactivity with antibodies against non-TP pathogens.

The detection accuracy of the SCM-based TP-Ab immunosensor was further assessed using a reference serum panel comprising 40 samples. Representative colorimetric results for these samples are shown in Fig. 5B. Notably, this TP-Ab immunosensor integrates ELISA principles for testing and result visualization, following the commonly used methodology for manual calculations to determine the cutoff value in ELISA assays. This value is calculated as the mean value of negative samples plus three times the standard deviation (34). The Corrected *I*_red_ signals from 28 negative samples from the banked sample and the negative control were analyzed. Based on these data, the crude cutoff value was determined to be 0.0944. Samples with Corrected *I*_red_ values exceeding 0.0944 were classified as positive, while those with values below 0.0944 were classified as negative. Statistical analysis of the 40 samples indicated that 19 samples had Corrected *I*_red_ values below the cutoff value, while the remaining 21 samples had values above it. The Corrected *I*_red_ values of the positive and negative controls are 0.391 and 0.044, respectively. Simultaneously, each sample was compared in parallel with the reference results tested by treponemal antibody detection methods (TP-ELISA). The results demonstrated good concordance between the TP-Ab immunosensor and the commercial kit. According to the crude cutoff value of 0.0944, sample 2, with a Corrected *I*_red_ value of 0.096, would have been classified as positive (since 0.096 > 0.0944), making the determined results of sample 2 inconsistent with the reference results. Therefore, it was necessary to find a more accurate method for calculating the optimal cutoff value. The determined results are shown in Table 1.

**TABLE 1:** The Corrected *I*_red_ values for reference serum panel and determined results^*a*^

Reference serum panel No.	TP-Immunosensor		TP-ELISA reference results	Reference serum panel No.	TP-immunosensor		TP-ELISA reference results
	Corrected *I*_red_ values	Determined results			Corrected *I*_red_ values	Determined results	
1	0.027	−	−	22	0.044	−	−
2	0.096	+	−	23	0.026	−	−
3	0.468	+	+	24	0.031	−	−
4	0.360	+	+	25	0.489	+	+
5	0.047	−	−	26	0.373	+	+
6	0.395	+	+	27	0.272	+	+
7	0.031	−	−	28	0.231	+	+
8	0.038	−	−	29	0.449	+	+
9	0.082	−	−	30	0.326	+	+
10	0.403	+	+	31	0.039	−	−
11	0.030	−	−	32	0.036	−	−
12	0.260	+	+	33	0.315	+	+
13	0.409	+	+	34	0.310	+	+
14	0.039	−	−	35	0.032	−	−
15	0.339	+	+	36	0.054	−	−
16	0.441	+	+	37	0.063	−	−
17	0.053	−	−	38	0.408	+	+
18	0.041	−	−	39	0.307	+	+
19	0.368	+	+	40	0.040	−	−
20	0.242	+	+	Pos	0.391	+	+
21	0.021	−	−	Neg	0.044	−	−

^*a*^ “+” indicates positive results, while “−” indicates negative results. The determined results of reference serum panel samples were based on the crude cutoff value of 0.0944.

### Comprehensive statistical analysis

**Fig 6:**
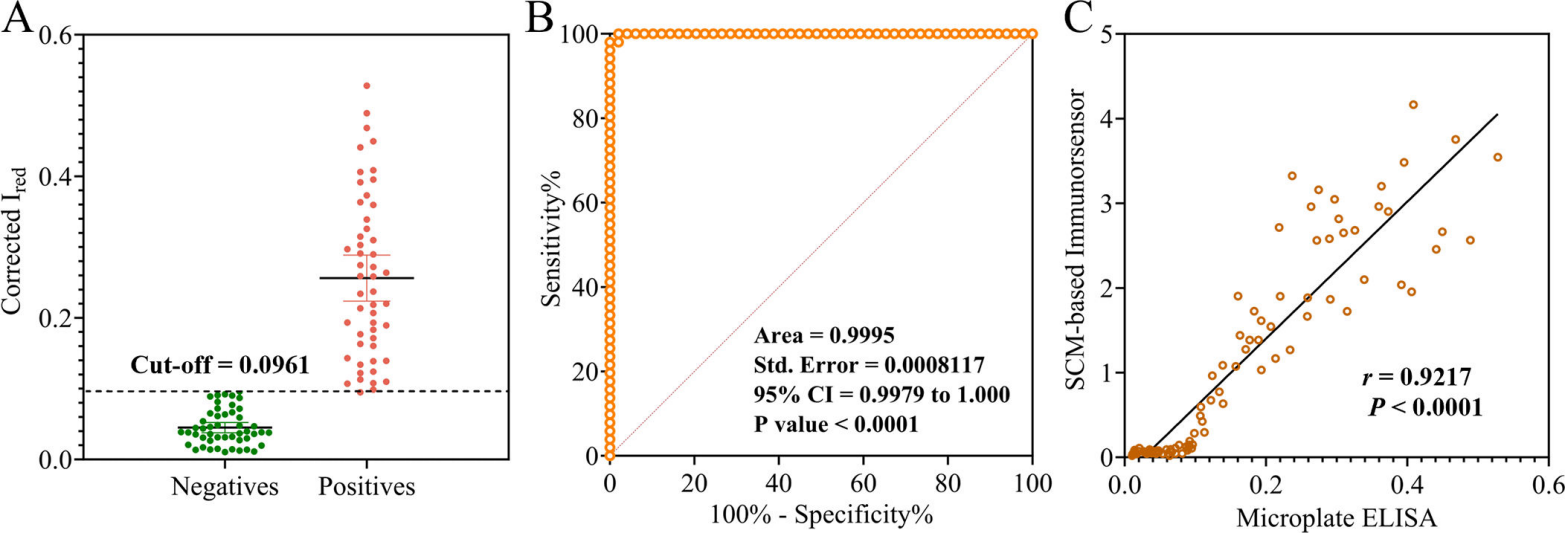
Comprehensive statistical analysis of reference serum panel and banked samples. (**A**) Corrected *I*_red_ values for the 100 samples. (**B**) ROC curve illustrating the diagnostic performance of the immunosensor. (**C**) Comparative statistical analysis of results from both methods.

To further validate the performance of the developed method, a comprehensive statistical analysis was conducted using data from 100 samples, including the 58 banked samples (30 positive and 28 negative), the reference serum panel comprising 40 samples (20 positive and 20 negative), and one positive and one negative control. Following color extraction and data curation, the Corrected *I*_red_ values for all 49 negative samples ranged from 0.016 to 0.0961, while those for 51 positive samples ranged from 0.0949 to 0.528 (Fig. 6A). The positive and negative controls exhibited Corrected *I*_red_ values of 0.50 and 0.04, respectively. The ROC analysis was performed as described in “Imaging, color extraction, data curation, and statistical analysis” to further assess assay performance. The analysis identified two potential cutoff values, 0.0921 and 0.0961, which closely aligned with the preliminary calculated crude cutoff value of 0.0944. These thresholds corresponded to sensitivities of 100% and 98.04%, and specificities of 97.96% and 100%, respectively (Table 2). Based on the Youden index, the cut off of 0.0961 (Youden index = 0.9804) was selected as the optimal cutoff value, which achieved 100% specificity, and it was superior to the cutoff value of 0.0921 (Youden index = 0.9796) with 97.96% specificity. This decision reflects the clinical imperative for high specificity in screening assays.

**TABLE 2:** Cutoff values with corresponding sensitivity, specificity, and 95% confidence interval (CI)

Criterion	Sensitivity	95% CI	Specificity	95% CI	+LR	−LR
≥0.010518096	100.00	93.0–100.0	0.00	0.0–7.3	1.00	NA^*a*^
>0.092102967	100.00	93.0–100.0	97.96	89.1–99.9	49.00	0.00
>0.09486634	98.04	89.6–100.0	97.96	89.1–99.9	48.04	0.020
>0.096107692	98.04	89.6–100.0	100.00	92.7–100.0	NA	0.020
>0.527883112	0.00	0.0–7.0	100.00	92.7–100.0	NA	1.00

^*a*^ "NA" indicates that these values are not applicable.

All Corrected *I*_red_ values for negative samples, except one, fell below the 0.0921 threshold, while all positive samples confirmed by TP-Ab immunosensor exhibited Corrected *I*_red_ values above 0.0921. This threshold successfully differentiated between positive and negative controls with sensitivity (98.04%) and maximum specificity (100%). Additionally, the ROC curve analysis also demonstrated excellent diagnostic performance, yielding an AUC of 0.9995, with a 95% confidence interval (CI) of 0.9979–1.00 (Fig. 6B), indicating near-perfect diagnostic accuracy. Finally, the SCM-based TP-Ab colorimetric immunosensor was compared with a commercial microplate ELISA kit. A statistically significant correlation was observed between the two methods (*r* = 0.9217, *P* < 0.0001) (Fig. 6C). Using pre-defined cutoff values of 0.0961 (SCM-based immunosensor) and 0.274 (microplate ELISA), both methods achieved 100% agreement in qualitative classification across 100 samples. These results confirm the reliability and robustness of the SCM-based TP-Ab immunosensor for diagnostic applications.

## DISCUSSION

Here, we present a novel colorimetric immunosensor based on SCM-integrated ELISA for the detection of TP antibodies. As a new type of biomaterial, the white-colored cocoons of *B. mori*, composed of immunogenic silk proteins, provide a natural free-standing substrate with a three-dimensional, multilayer, and porous structure, without the need for any chemical regeneration of the membranes (37–39). This superior physical structure provides a larger surface area and a greater number of available binding sites for the immobilization of protein molecules. The binding interactions on the SCMs are driven by immune affinity coupling between antigens and antibodies, which enhances the specificity of the coupling and facilitates sensor activation and functionalization. In contrast to nonspecific physical adsorption and chemical modification on polystyrene or other synthetic substrates, this affinity-based binding improved the directionality, stability, and sequential interaction between molecular components (40, 41), thereby contributing to the enhanced performance of the TP-Ab immunosensor. As a result, the SCM-based TP-Ab immunosensor exhibits a pronounced colorimetric signal variation even at low antibody concentrations, demonstrating a lower limit of detection while maintaining reliable performance at higher concentrations. These characteristics underscore the superior sensitivity of the SCM-based TP-Ab immunosensor for detecting treponemal antibody. The 3H12 antibody, developed in our laboratory against the TP47 antigen, was immobilized onto the functional SCM, specifically binding to TP47 antigenic sites. TP99, a chimera antigen containing TP15, TP17, and TP47 epitopes as recognition elements, was used as the capture antigen by binding with 3H12 in the TP-Ab immunosensor. The inclusion of these TP99 epitopes allowed broader binding to TP-Ab, enhancing both specificity and detection accuracy (42).

This TP-Ab immunosensor integrates ELISA principles for testing and result visualization, achieving a notable improvement without the need for specialized instruments. Only after smartphone camera imaging, the color extraction, data curation, and statistical analysis provide more accurate results. By comparing the primary color channels, the red channel demonstrated the highest color variability, indicating superior signal sensitivity, and the Corrected *I*_red_ values were selected as the primary analytical signal, calculated using equation 2. In addition to determining the results through color extraction analysis, rapid and preliminary judgments can also be made by visually observing the colorimetric signal variation with the naked eye: negative samples display white color, representing SCM’s intrinsic color, while positive reactions exhibit a strong blue contrast. Therefore, the SCM-based TP-Ab immunosensor shows potential for use in various diagnostic scenarios, from standard clinical laboratories to resource-limited settings, particularly for rapid small-scale or point-of-care testing.

The SCM-based TP-Ab immunosensor (F-SCMs/3H12/TP99) was fabricated with the optimal immobilization concentrations of 3H12 and TP99. This immunosensor demonstrated a detection limit of 0.48 mIU/mL, significantly surpassing the 0.68 mIU/mL limit of commercial microplate ELISA kits, and showed no cross-reactivity with antibodies against HBV, HCV, and HIV-1. These antibodies of pathogens were selected for this initial specificity study because they are included in the mandatory blood donor screening panel in China and have been reported to occasionally cause false-positive results in syphilis serological assays, possibly due to atypical antibody responses (43). While this evaluation provides a foundational assessment of assay specificity, further studies will be conducted to assess potential cross-reactivity with antibodies against phylogenetically or antigenically related microorganisms, including *Borrelia* and *Leptospira* species, other gram-negative bacteria, and closely related *Treponema* species. This ROC-derived optimal cutoff value of 0.0961 was ultimately used for all subsequent classification of clinical samples, prioritizing 98.04% sensitivity and 100% specificity, critical for minimizing false-negative results and reducing the risk of transmission in the context of infectious diseases. The immunosensor demonstrated high overall accuracy, with an AUC of 0.9995 ± 0.0008 and a 95% CI of 0.9979–1.00. A statistically significant correlation was observed between the TP-Ab immunosensor and the microplate ELISA (*r* = 0.9217, *P* < 0.0001), with 100% agreement. These results confirm the reliability and robustness of the SCM-based TP-Ab immunosensor for diagnostic applications.

Although the TP-Ab immunosensor demonstrated promising diagnostic performance in this preliminary study, one major limitation of this study is the lack of sufficient clinical sample data from multiple time points or different stages of syphilis. Future large-scale clinical validation involving more samples is necessary to ensure statistical robustness and enhance the broader applicability of the product in diverse clinical settings. Previous studies have shown that recombinant TP antigens can enhance treponemal test performance, with IgG or IgM antibodies against TP15, TP17, and TP47 detectable 2–4 weeks after infection, earlier than non-treponemal antibodies (12, 44). However, further validation is needed to confirm that the chimeric recombinant antigen TP99, containing these epitopes, can accurately detect IgG or IgM antibodies specific to syphilis and thus demonstrate the broader applicability of the TP-Ab immunosensor. In addition, the natural SCMs were manually screened to ensure consistency in small-scale experiments; their inherent variability remains a challenge for reproducibility and scalability. Future efforts will focus on reproducible matrix fabrication using engineered silk fiber paper, as reported in our previous study (45), which could be used for the detection of TP antibodies.

### Conclusions

In summary, we have developed a novel SCM-based immunosensor for the detection of TP antibodies, representing the first application of an SCM-derived colorimetric immunosensor integrated ELISA. The optimized protocol for detecting TP antibodies in human serum has been successfully demonstrated, highlighting the sensor’s potential for on-site syphilis diagnosis. Despite its promising performance, further optimization of SCM production to enhance reproducibility and scalability and expansion to detect other biomarkers are necessary for broader applicability, especially in point-of-care testing in low-resource settings. This work paves the way for the development of cost-effective, SCM-based, smartphone-readable immunosensors using natural substrates. The SCM-based TP-Ab immunosensor holds substantial potential for early syphilis screening and diagnosis, particularly in resource-limited or remote areas, addressing critical gaps in accessible healthcare diagnostics.

## SUPPLEMENTAL MATERIAL

Figure S1 spectrum.00094-25-s0001.docx Reference serum info.

Figure S2 spectrum.00094-25-s0002.docx Colorimetric workflow.

ASM does not own the copyrights to Supplemental Material that may be linked to, or accessed through, an article. The authors have granted ASM a non-exclusive, world-wide license to publish the Supplemental Material files. Please contact the corresponding author directly for reuse.
