# Supplementary material for: A novel colorimetric immunosensor based on silk cocoon membrane-integrated ELISA for treponemal antibody detection
Source: Microbiol Spectr. 2025 Jul 22;13(9):e00094-25. doi: 10.1128/spectrum.00094-25 (PMC12403623; doi:10.1128/spectrum.00094-25)
Supplement: Figure S1 — Reference serum info. [file spectrum.00094-25-s0001.docx]

**Supplementary Material**

**A Novel Colorimetric Immunosensor based on Silk Cocoon Membrane-integrated ELISA for Treponemal Antibody Detection**


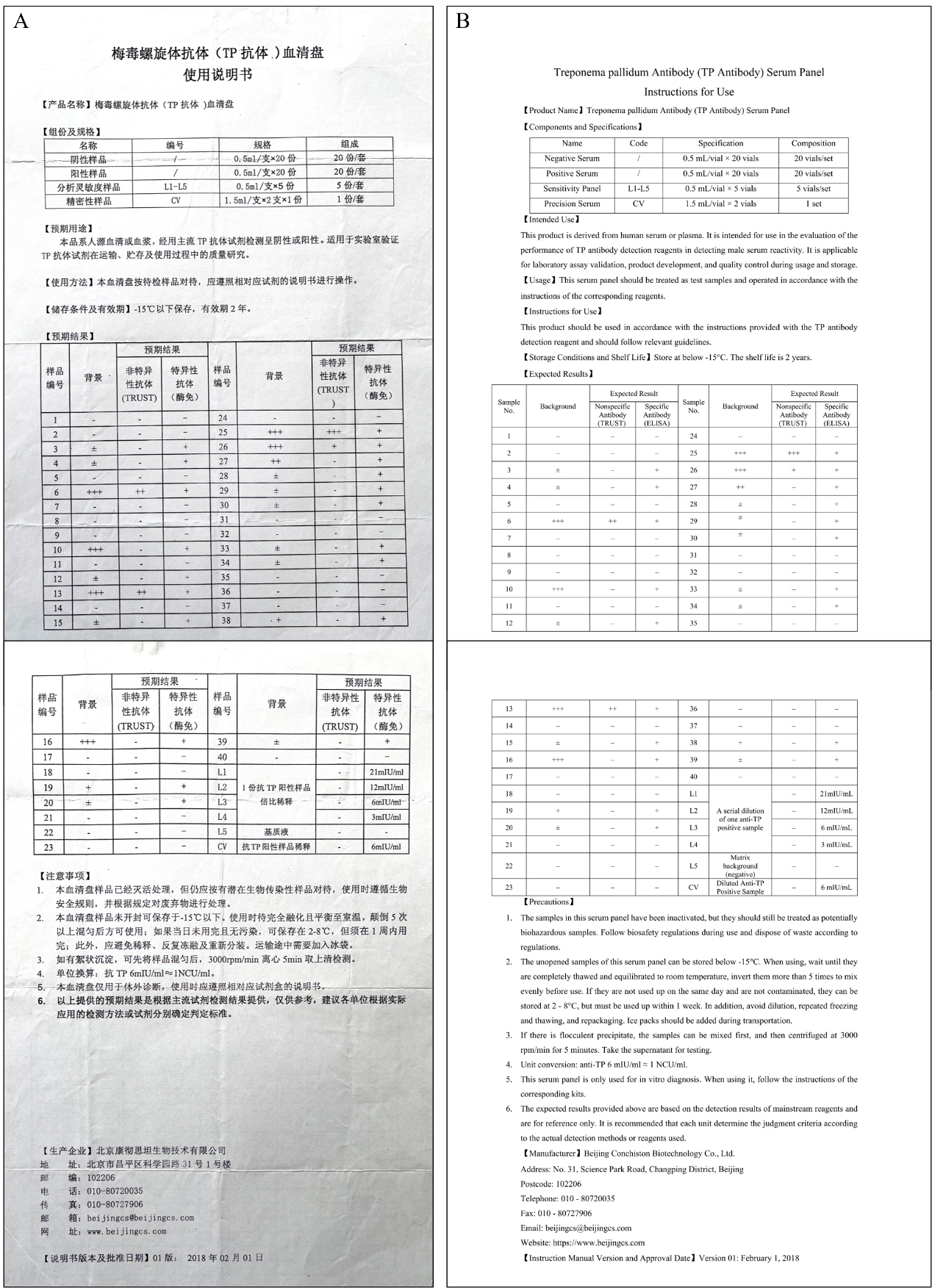


FIG S1. The detailed information about reference serum panel and standard TP-Ab serum. (A) Chinese version; (B) English version.
