# Supplementary material for: A novel colorimetric immunosensor based on silk cocoon membrane-integrated ELISA for treponemal antibody detection
Source: Microbiol Spectr. 2025 Jul 22;13(9):e00094-25. doi: 10.1128/spectrum.00094-25 (PMC12403623; doi:10.1128/spectrum.00094-25)
Supplement: Figure S2 — Colorimetric workflow. [file spectrum.00094-25-s0002.docx]

**Supplementary Material**

**A Novel Colorimetric Immunosensor based on Silk Cocoon Membrane-integrated ELISA for Treponemal Antibody Detection**


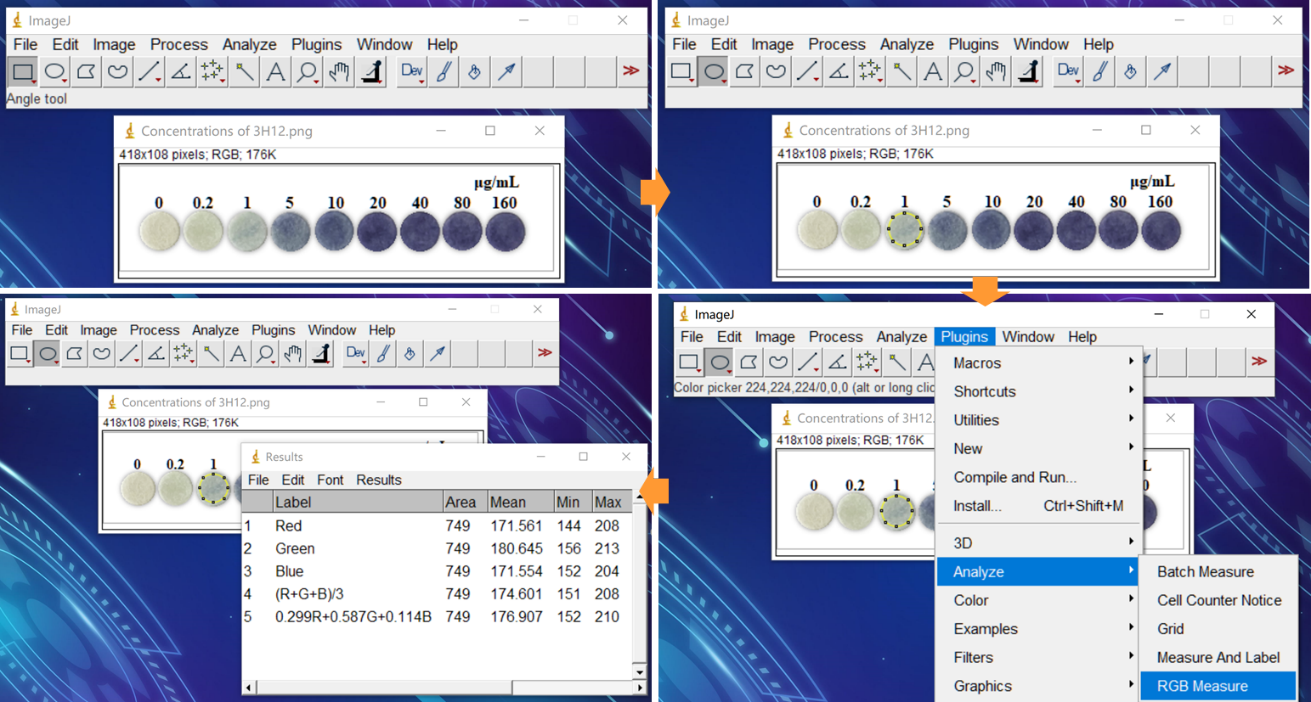


FIG S2. Analysis step for the colorimetric measurements of smartphone image J software.
